# Knowledge and attitudes on medication adherence and residual symptoms in individuals with depression: a survey at a University Hospital

**DOI:** 10.1186/s12888-023-04706-y

**Published:** 2023-03-29

**Authors:** Jarurin Pitanupong, Jakkapon Sammathit

**Affiliations:** Department of Psychiatry, Faculty of Medicine, Prince of Songkla University, Hat Yai, Songkhla, 90110 Thailand

**Keywords:** Attitude, Depression, Knowledge, Medication adherence, Residual symptom

## Abstract

**Background:**

Depression is a common disease and the relapse of depression can cause functional impairment. Good medication adherence and relapse prevention should be targeted to achieve normal functioning. This study aimed to evaluate the levels of knowledge, attitude toward depression, and medication adherence among individuals with depression.

**Methods:**

A cross-sectional study surveyed Thai individuals with depression at the psychiatric outpatient clinic of Songklanagarind Hospital; from April to August 2022. The questionnaires inquired about:1) demographic information, 2) knowledge and attitude toward depression questionnaire, 3) the medication adherence scale in Thais (MAST), 4) the Patient Health Questionnaire-9 (PHQ-9), 5) the stigma questionnaire, 6) a patient-doctor relationship questionnaire (PDRQ-9), and 7) the Revised Thai Multidimensional Scale of Perceived Social Support (rMSPSS). All data were analyzed using descriptive statistics. Chi-square or Fisher’s exact test, and Wilcoxon rank sum test were used.

**Results:**

Of all 264 participants, the majority of them were female (78.4%). The overall mean age was 42.3 ± 18.3 years. Most participants had good knowledge and a positive attitude regarding any relationship problems, childhood trauma or bad memories, or having a chemical imbalance in the brain as one of the main causes that result in depression (86.4, 82.6, 77.3%, respectively). They disagreed with common stereotypical assumptions towards individuals with depression. Most of them had good medication adherence (97.0%), low or no level of stigma (92.5%), high perceived social support from family (64.4%), and good doctor-patient relationships (82.2%). Due to most participants reporting having good medication adherence, then an attempt to indicate the factors associated with medication adherence could not be established in this study. This study found that individuals reporting residual symptoms of depression had higher levels of knowledge and perceived stigma, but lower levels of family support compared to those without residual symptoms.

**Conclusion:**

Most participants reported good knowledge and a positive attitude toward depression. They exhibited good medication adherence, a low level of stigma, and a high level of social support. This study revealed a correlation between the presence of residual symptoms of depression and increased levels of knowledge, perceived stigma, and reduced family support**.**

## Background

Major depressive disorder (MDD) is a significant mental health problem throughout the world. Untreated MDD may result in progressive alterations in brain morphometry that can cause chronicity, recurrence, and dysfunction and constitutes both economic and social burdens for families, communities, and societies [1]. Nowadays, evidence indicates that the dysregulation of neurotransmitters; depletion of serotonin, norepinephrine, and dopamine plays a major role in MDD [2]. Therefore, pharmacological treatment is necessary and effective in the treatment of MDD [3]. The effectiveness of antidepressants in treating MDD is hindered by poor medication adherence, which can impede sustained remission and functional restoration [4]. Patients who do not adhere to their medication regimen have been found to experience worse clinical outcomes, including increased MDD severity, emergency department visits, hospitalization, higher risks of relapse and recurrence, decreased treatment response, and lower remission rates [5]. Improving adherence to antidepressant medication is therefore a critical aspect in the management of MDD [4, 6]

Previous studies have reported that a significant proportion of individuals with MDD, ranging from 60.0–70.0%, have poor medication adherence [7, 8]. Understanding MDD and its treatment may have a significant impact on adherence to antidepressants [9]. Factors that contribute to poor medication adherence include high severity of illness, self-stigma, and negative attitudes among patients and caregivers towards MDD [8, 10]. This is due to the fact that individuals with MDD may experience distress from rejection and discrimination, leading to reduced self-esteem and life satisfaction [10]. Additionally, older adults with MDD are found to have a high level of public stigma and are less likely to seek mental health management [11].

Previous studies have identified that older age, male gender, lower educational level, and living alone are associated with personal and perceived stigma toward MDD. These factors can negatively impact the intention of individuals with MDD to seek professional help [12]. On the other hand, factors such as younger age, female gender, employment, a family history of MDD, and higher education are associated with better attitudes and beliefs toward antidepressants and MDD among individuals with chronic depression [13]. Additionally, it has been previously stated that lower knowledge and a prejudiced view toward MDD can contribute to poor medication adherence among individuals with MDD. Such poor adherence can result in inadequate treatment, treatment resistance [14, 15], and the presence of residual symptoms of MDD [16].

A previous cross-cultural study has shown that non-Western countries tend to have higher levels of stigma towards mental illnesses compared to Western countries [17]. Developed countries generally have lower levels of stigmatization toward mental illnesses than underdeveloped countries [18]. Additionally, this stigmatization towards MDD tends to be higher in Eastern compared to Western countries [17, 18]. A study on older Chinese individuals (aged over 60 years) with MDD found that low medication adherence was prevalent, with only 51 (37.8%) of the 135 participants having high adherence. Additionally, the study found that beliefs about medication were a significant factor affecting adherence, with "forgetting to take the medicine" and "feeling better and stopping medication" being the most common reasons for non-adherence [19].

Few studies have been conducted in Thailand on the understanding and attitudes toward MDD. One recent study examined the connection between depression literacy and stigma among individuals with MDD [20]. Another study examined the attitudes and stigmas toward MDD among the general population in different regions of Thailand. This study found that the highest level of stigma was present in the western region. Factors associated with higher levels of prejudice included male gender, younger age, higher educational level, and lack of experience with individuals with mental illnesses [21]. Moreover, factors within society and among family members can contribute to individuals with MDD feeling shame, and unable to accept themselves. These can negatively impact seeking treatment among individuals with MDD [11].

As mentioned above, a previous study has shown that there are variations in knowledge and attitudes toward MDD among different regions in Thailand [21]. While the majority of the Thai population is Buddhist, many provinces in the southern region are predominantly Muslim. Therefore, southern peoples’ beliefs may differ from those of Thais from other regions. To our knowledge, no study on knowledge or attitude toward MDD and medication adherence has been conducted in the southern region of Thailand over the past decade. Therefore, this study aimed to evaluate the levels of knowledge, attitudes toward MDD, and medication adherence among individuals with MDD at Songklanagarind hospital, which is an 800-bed university hospital serving as a tertiary referral center in Southern Thailand. Furthermore, a previous study conducted at Songklanagarind hospital found that nearly half (45.4%) of individuals with MDD, who received antidepressants for more than 12 weeks, still had symptoms of MDD [22]. Therefore, the correlation between the levels of knowledge, attitudes toward MDD including the other factors (demographic data, level of stigma, and perceived social support) and the presence of residual symptoms of MDD were also evaluated.

## Methods

After being approved by the Ethics Committee of the Faculty of Medicine, Prince of Songkla University (REC: 65–026-3–4), this cross-sectional study was conducted at the psychiatric outpatient clinic, at Songklanagarind Hospital. All outpatients with MDD, who received antidepressants for more than 12 weeks, had an appointment, and were followed up regularly at psychiatric outpatient clinic; from April to August 2022, were invited to participate in the study**.** This study involved participants who had been diagnosed with MDD and received at least 12 weeks of consistent antidepressant therapy. It is known that it can take up to 12 weeks for some patients to achieve complete remission [23, 24].

As per the literature review, 25.0–45.0% and 60.0% of the general population had poor knowledge of MDD and prejudice attitudes toward MDD, respectively [11, 25]. In addition, 60.0–70.0% of individuals with MDD were reported to be medication non-adherent [7, 8]. Then the researcher used these towards the sample size calculation to estimate the proportion in this study. The command ‘n.for.survey’ in the Epicalc package in the R program was used (given delta = 0.06 and alpha = 0.05). Therefore, the required sample size for our study was 264.

Based on the following criteria: ICD-10 code; F33.0-F33.9; except F33.3, individuals with MDD, as diagnosed by their psychiatrists, were selected in the medical register. Those who acknowledged their diagnosis, aged more than 20 years, used of Thai language, agreed to collaborate, and completed all parts of the questionnaires were included. Meanwhile, those who were unaware of their diagnosis, had comorbidity or more than one psychiatric diagnosis, had active substance usage, presented psychotic symptoms, did not decide to collaborate or wished to withdraw from the study, and lacked the mental capacity to complete all of the questionnaires, were excluded.

All of the individuals with MDD were approached by the research assistant for recruitment in the study. An information sheet, which described the allotted time to complete the questionnaires and the rationale for the study was distributed to them. All participants had at least 20 min to deliberate whether to collaborate in the study or not. Participants willing to collaborate were invited to a private room to complete the questionnaires. They were announced that they could withdraw at any time if they felt distressed or worried without providing any reason.; and with no impact on their regular treatment. Furthermore, if the participants exhibited a high level of distress, recommendation and/or further clinical intervention were provided to them. Moreover, for protecting the participants’ identities, they were informed that there was no requirement for their signatures and that their information would remain anonymous.

### Questionnaires

- 1. General demographic information inquired around areas related to age, gender, marital status, religion, educational level, occupation, income, and history of psychiatric hospitalization.
- 2. Knowledge and attitude toward depression questionnaire, a self-rating questionnaire developed from the English version of the previous questionnaire and by referring to previous literature. The questionnaire mainly comprised 16 questions. The first section, of nine items, mainly focused on the evaluation of the perception of the causes of depression, and the rest section consisted of seven items exploring attitudes toward people with depression. The score of each question was rated as agreeing, doubtful, and disagreeing. The questionnaire demonstrated Cronbach’s alpha coefficient of 0.71 [25, 26]. The Thai version of the questionnaire underwent forward and backward translation, as well as content validity assessment by five psychiatrists. The resulting content validity index (CVI) score was 0.8.
- 3. Medication adherence scale in Thais (MAST), a self-rating questionnaire which comprised 8 questions. The questionnaire assessed the frequency of medication adherence behaviors over the past month, including forgetting to take medication, not taking medication at all meals, not taking medication at the scheduled time, altering the dosage, not taking all prescribed medications, failing to attend scheduled follow-ups, and a lack of medication. The score of each question was rated on a 0–5 scale; 0 (never); 1 (1–2 times/month); 2 (3–5 times/month); 3 (6–9 times/month); 4 (10–15 times/month); 5 (> 15 times/month). The total score was summed to range from 0 to 40. The cut-off point of the MAST was 34, the total score of 34 or more was poor medication compliance. The questionnaire demonstrated a sensitivity of 85.8; and a specificity of 89.7; a positive predictive value of 90.6; a negative predictive value of 84.7; Cronbach’s alpha coefficient of 0.828 [27]. The MAST has been utilized as a tool for measuring medication adherence in individuals with diabetes [27], hypertension [28], and psychiatric disorders [29, 30].
- 4. The Patient Health Questionnaire-9 (PHQ-9) Thai version, a self-rating questionnaire to evaluate depression comprising of 9 questions, employed a 4-point rating scale; 0 (never); 1 (rarely); 2 (sometimes); 3 (always). The total score was summed to range from 0 to 27; 0–4 (no/minimal depression); 5–9 (mild depression); 10–14 (moderate depression); 15–19 (moderately severe); 20–27 (severe depression). The questionnaire demonstrated a sensitivity of 0.84; specificity of 0.77; Cronbach's alpha coefficient of 0.79. The Thai version of the PHQ-9 had acceptable psychometric properties for the screening of major depression with a recommended cut-off score of nine or greater [31]. In this study, individuals with MDD who had received antidepressant therapy for more than 12 weeks and still had a PHQ-9 score of nine or greater were identified as having residual symptoms of MDD [22–24].
- 5. The stigma questionnaire Thai version, a self-rating questionnaire to evaluate two domains of stigma for psychiatric patients; being stigmatized and being separated. It comprised 21 questions and employed a 6-point rating scale; 0 (never); 1 (rarely); 2 (once in a while); 3 (sometimes); 4 (often); 5 (very often). The 4 items must be reversed before totaling. The total score was summed to range from 0 to 105; 0–21 (no stigma); 22–43 (low level of stigma); 44–65 (moderate level of stigma); 66–87 (high level of stigma); 88–105 (very high level of stigma). The questionnaire demonstrated Cronbach's alpha coefficient of 0.76; the CVI score was 0.9 [32, 33].
- 6. A patient-doctor relationship questionnaire (PDRQ-9) Thai version, a self-rating questionnaire which comprised of 9 questions and employed a 5-point rating scale; 1(not at all appropriate); 2 (somewhat appropriate); 3 (appropriate); 4 (mostly appropriate); 5 (totally appropriate). The total score was summed to range from 9 to 45; 36 or higher (good doctor-patient relationship); 18–35 (moderate doctor-patient relationship); 17 or lower (poor doctor-patient relationship) [34]. The questionnaire demonstrated Cronbach's Alpha coefficient of 0.7–0.94 [35, 36]. In this study, the Thai version of the questionnaire was used, which was translated from the English version. The translation and content validity were evaluated by five psychiatrists, resulting in a CVI score of 0.8.
- 7. The Revised Thai Multidimensional Scale of Perceived Social Support (rMSPSS), a self-rating questionnaire to measure the extent to which an individual felt supported by family members, friends, and significant others. It comprised 12 questions and employed a 7-point rating scale; 1(less agree); 7 (the most agree). The score was divided into 3 groups including the significant other subgroups (questions 1, 2, 5, 10), family subgroup (questions 3, 4, 8, 11), and friend subgroup (questions 6,7,9,12). The total score was summed to range from 12 to 84. The mean of each subscale ranged from 1 to 7; 1–2.9 (low support); 3–5 (moderate support); 5.1–7 (high support). The questionnaire demonstrated Cronbach's alpha coefficient of 0.87 [37, 38].

### Statistical analysis

All data were analyzed to describe the knowledge, attitude toward MDD, and medication adherence using descriptive statistics. This was calculated using frequency, proportions, means (standard deviation, SD), and median (interquartile range, IQR) for patient demographics. Chi-square or Fisher’s exact test, and Wilcoxon rank sum test were used for comparison between the group.

## Results

### Demographic characteristics

From April to August 2022, 264 individuals with MDD attended psychiatric outpatient clinic, and all of them agreed to collaborate and complete the questionnaires. The majority of them were female (78.4%), Buddhist (82.2%), single/divorced (59.1%), and bachelor’s degree or above (62.5%) (Table 1). The mean age was 42.3 ± 18.3 years (20–84 years old), and the median (IQR) age was 40.5 (24.0, 59.0). The median (IQR) income was 15,000 (8,000, 30,000) baths per month (moderate income).

**Table 1:** Demographic characteristics (*N* = 264)

Demographic characteristics	Number (%)
**Gender**	
Male	57 (21.6)
Female	207 (78.4)
**Marital Status**	
Single/Divorce	156 (59.1)
Married	108 (40.9)
**Religion**	
Buddhism	217 (82.2)
Islam/Christianity/Other	47 (17.8)
**Education level**	
Primary school or below	32 (12.1)
Secondary school	45 (17.0)
Diploma	22 (8.3)
Bachelor’s degree or above	165 (62.5)
**Occupation**	
Government officer/ state enterprise employee / company employee	82 (31.1)
Self-employed / merchant/ personal business/ agriculture	67 (25.4)
Student	61 (23.1)
Unemployed	54 (20.5)
**Perceived relationship of family**	
Good	157 (59.5)
Poor	30 (11.4)
Neutral/Other	77 (29.2)
**Having physical illness**	
No	138 (52.3)
Yes	126 (47.7)
**Number of depressive episode**	
One	227 (86.0)
Two	28 (10.6)
Three or more	8 (3.0)
No answer	1 (0.4)
**History of psychiatric hospitalization**	
No	215 (81.4)
Yes	49 (18.6)

### Depression profile and medication adherence

The median age (IQR) of MDD onset was 33.5 (22.0, 49.0) years. Most participants (86.0%) reported having one episode of MDD, and no history of psychiatric hospitalization (81.4%) (Table 1). All participants received antidepressants for at least 12 months, with a median treatment duration (IQR) of 29.0 (12.0, 72.0) months. All of them still received antidepressants, had an appointment, and were followed up regularly at the psychiatric clinic. Out of all participants, 54.5% had a PHQ-9 score of nine or greater. Loss of interest (72.2%), feeling down (72.2%), and sleep disturbance (70.8%) were the most residual symptoms of MDD. Additionally, most of them reported good medication adherence (97.0%), and a good doctor-patient relationship (82.2%) (Table 2).

**Table 2:** The score of PHQ-9, medication adherence, doctor-patient relationship, and stigma (*N* = 264)

Type of score	Number (%)
**PHQ-9 score**	
Minimal/ mild	132 (50.0)
Moderate	52 (19.7)
Moderately severe	39 (14.8)
Severe	41 (15.5)
Mean (SD)	10.7 (7.4)
Median (IQR)	9.5 (4.0, 16.3)
**Medication adherence**	
Good	256 (97.0)
Poor	3 (1.1)
No answer	5 (1.9)
Mean (SD)	5.7 (7.0)
Median (IQR)	3.0 (1.0, 8.0)
**Doctor-patient relationship**	
Good	217 (82.2)
Moderate	45 (17.0)
Poor	2 (0.8)
Mean (SD)	39.2 (5.3)
Median (IQR)	39 (36, 45)
**Stigma**	
No	49 (18.6)
Low	195 (73.9)
Moderate	18 (6.8)
High	2 (0.8)
Mean (SD)	29.2 (9.5)
Median (IQR)	28 (23, 34)

*IQR* Interquartile range, *SD* Standard deviation

### Knowledge and attitude toward depression

Most participants reported a score of good knowledge toward MDD and that marital or relationship problems were one of the main causes that resulted in MDD, as well as childhood trauma or bad memories of the past, or having a chemical imbalance in the brain (86.4, 82.6,77.3%, respectively). According to attitudes toward MDD, they reported disagreeing with the concept that individuals with MDD were crazy, not friendly, and dangerous (90.2, 85.2, 83.3%, respectively) (Table 3).

**Table 3:** Knowledge and attitude toward depression (*N* = 264)

**Knowledge toward depression**	**Number (%)**		
	**Agree**	**Disagree**	**Unsure**
1) Genetic is one of the main factors of contributing major depressive disorder	103 (39.0)	111 (42.0)	50 (18.9)
2) A chemical imbalance in the brain is one of the possible causes of major depressive disorder	204 (77.3)	26 (9.8)	34 (12.9)
3) Lack of social support contributes a lot to the occurrence in major depressive disorder	185 (70.1)	57 (21.6)	22 (8.3)
4) Martial/relationship problem is one of the main causes that result in major depressive disorder	228 (86.4)	22 (8.3)	14 (5.3)
5) Frequent alcohol/drug abuse lead to a major depressive disorder	93 (35.2)	79 (29.9)	92 (34.8)
6) Childhood trauma or bad memories of the past can lead to major depressive disorder	218 (82.6)	34 (12.9)	12 (4.5)
7) Financial problems have a contribution to resulting major depressive disorder	187 (70.8)	50 (18.9)	27 (10.2)
8) Major depressive disorder is due to supernatural and spiritual reasons	56 (21.2)	156 (59.1)	52 (19.7)
9) Casting black magic on someone may result in major depressive disorder	25 (9.5)	191 (72.3)	48 (18.2)
**Attitude toward depression**			
1) Individuals with major depressive disorder are crazy	13 (4.9)	238 (90.2)	13 (4.9)
2) Individuals with major depressive disorder are dangerous	24 (9.1)	220 (83.3)	20 (7.6)
3) Individuals with major depressive disorder are not friendly	21 (8.0)	225 (85.2)	18 (6.8)
4) Individuals with major depressive disorder are unpredictable and can result in harm	93 (35.2)	133 (50.4)	38 (14.4)
5) Individuals with major depressive disorder are moody	125 (47.3)	91 (34.5)	48 (18.2)
6) Individuals with major depressive disorder are kind	140 (53.0)	45 (17.0)	79 (29.9)
7) Individuals with major depressive disorder have disturbing/negative thoughts; therefore, it’s better to avoid them	51 (19.3)	170 (64.4)	43 (16.3)

### Level of stigma and perceived social support

**Fig. 1:**
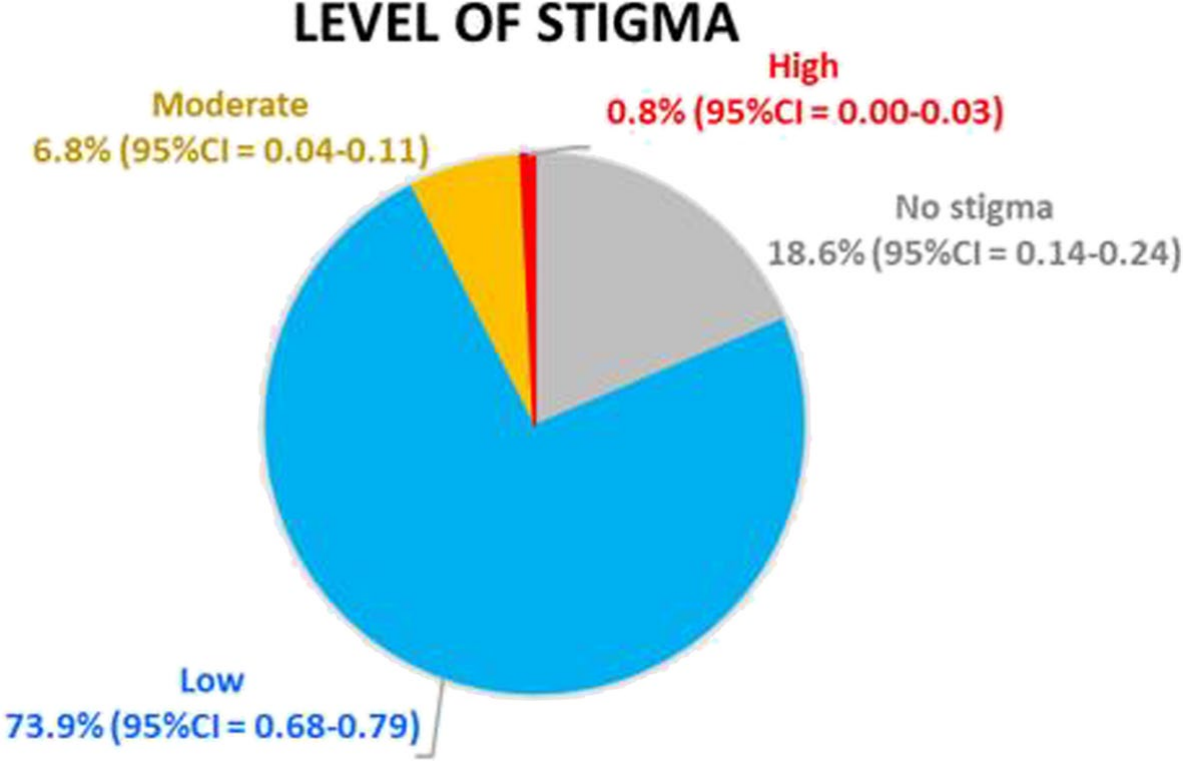
Level of stigma using the stigma questionnaire Thai version (*N* = 264)

Most participants reported low or no levels of stigma (73.9% and 18.6%, respectively) (Table 2). Only two participants (0.8%) reported a high level of stigma (Fig. 1). Moreover, more than half of the participants reported a high level of perceived support from family and friends (64.4% and 53.4%, respectively). Almost half of all participants (49.2%) reported a moderate level of perceived support for the significant other subscales (Table 4).

**Table 4:** The revised thai multidimensional scale of perceived social support (rMSPSS) (*N* = 264)

Perceived social support	Number (%) Level of social support				Mean (SD)	Median (IQR)
	**Low**	**Moderate**	**High**	**No answer**		
Family subscale	15 (5.7)	78 (29.5)	170 (64.4)	1 (0.4)	5.4 (1.4)	5.7 (4.5, 6.2)
Friend subscale	15 (5.7)	108 (40.9)	141 (53.4)	0 (0.0)	5.1 (1.3)	5.2 (4.0, 6.0)
Other subscale	20 (7.6)	130 (49.2)	114 (43.2)	0 (0.0)	4.8 (1.3)	4.7 (4.0, 6.0)

*IQR* Interquartile range, *SD* Standard deviation

#### Association between levels of knowledge and attitudes toward depression, level of stigma, doctor-patient relationship, perceived social support, and medication adherence

Because most participants (97.0%) reported having good medication adherence, it was not possible to explore if there is any association between the factors and medication adherence, in this study. Therefore, we tried to correlate the levels of knowledge and attitudes toward depression with the other variable factors including demographic characteristics.

In this study, there was a significant difference between the demographic characteristics; the perceived relationship of family, having a physical illness, with the doctor-patient relationship, and the presence of residual symptoms of MDD (Table 5).

**Table 5:** Association between demographic characteristics with the presence of residual symptoms, doctor-patient relationship and medication adherence

**Demographic Data**	Total	**Residual symptoms**		x^2^-test *P* value	**Doctor-patient relationship**		x^2^-test *P* value	**Medication adherence**		Fisher's exact test *P* value
		PHQ < 9	PHQ ≥ 9		Poor-moderate	Good		Good	Poor	
	*N* = 264	*N* = 120	*N* = 144		*N* = 47	*N* = 217		*N* = 256	*N* = 3	
**Gender**				0.168			0.890			0.520
Male	57 (21.6)	31 (25.8)	26 (18.1)		11 (23.4)	46 (21.2)		55 (21.5)	1 (33.3)	
Female	207 (78.4)	89 (74.2)	118 (81.9)		36 (76.6)	171 (78.8)		201 (78.5)	2 (66.7)	
**Marital Status**				< 0.001			0.223			0.272
Single/Divorce	156 (59.1)	55 (45.8)	101 (70.1)		32 (68.1)	124 (57.1)		150 (58.6)	3 (100)	
Married	108 (40.9)	65 (54.2)	43 (29.9)		15 (31.9)	93 (42.9)		106 (41.4)	0 (0.0)	
**Religion**				0.212			0.956			0.453
Buddhism	217 (82.2)	103 (85.8)	114 (79.2)		38 (80.9)	179 (82.5)		210 (82.0)	2 (66.7)	
Islam/Christianity/Other	47 (17.8)	17 (14.2)	30 (20.8)		9 (19.1)	38 (17.5)		46 (18.0)	1 (33.3)	
**Education level**				0.024			0.266			0.761
Primary school or below	32 (12.1)	18 (15.0)	14 (9.7)		2 (4.3)	30 (13.8)		32 (12.5)	0 (0.0)	
Secondary school	45 (17.0)	26 (21.7)	19 (13.2)		7 (14.9)	38 (17.5)		43 (16.8)	1 (33.3)	
Diploma	22 (8.3)	13 (10.8)	9 (6.2)		4 (8.5)	18 (8.3)		22 (8.6)	0 (0.0)	
Bachelor’s degree or above	165 (62.5)	63 (52.5)	102 (70.8)		34 (72.3)	131 (60.4)		159 (62.1)	2 (66.7)	
**Occupation**				< 0.001			0.235			0.371
Government officer/ state enterprise employee / company employee	82 (31.1)	44 (36.7)	38 (26.4)		18 (38.3)	64 (29.5)		79 (30.9)	1 (33.3)	
Self-employed / merchant/ personal business/ agriculture	67 (25.4)	35 (29.2)	32 (22.2)		11 (23.4)	56 (25.8)		66 (25.8)	0 (0.0)	
Student	61 (23.1)	9 (7.5)	52 (36.1)		13 (27.7)	48 (22.1)		57 (22.3)	2 (66.7)	
Unemployed	54 (20.5)	32 (26.7)	22 (15.3)		5 (10.6)	49 (22.6)		54 (21.1)	0 (0.0)	
**Perceived relationship of family**				0.004			0.057			0.691
Good	157 (59.5)	82 (68.3)	75 (52.1)		21 (44.7)	136 (62.7)		149 (58.2)	3 (100)	
Poor	30 (11.4)	6 (5.0)	24 (16.7)		6 (12.8)	24 (11.1)		30 (11.7)	0 (0.0)	
Neutral/Other	77 (29.2)	32 (26.7)	45 (31.2)		20 (42.6)	57 (26.3)		77 (30.1)	0 (0.0)	
**Having physical illness**				0.002			0.056			0.248
No	138 (52.3)	50 (41.7)	88 (61.1)		31 (66.0)	107 (49.3)		132 (51.6)	3 (100)	
Yes	126 (47.7)	70 (58.3)	56 (38.9)		16 (34.0)	110 (50.7)		124 (48.4)	0 (0.0)	
**Number of depressive episode**				0.426			1			0.355
One	227 (86.0)	100 (84.0)	127 (88.2)		40 (87.0)	187 (86.2)		221 (86.7)	2 (66.7)	
Two or more	36 (13.6)	19 (16.0)	17 (11.8)		6 (13.0)	30 (13.8)		34 (13.3)	1 (33.3)	
**History of psychiatric hospitalization**				0.942			0.182			0.468
No	215 (81.4)	97 (80.8)	118 (81.9)		42 (89.4)	173 (79.7)		208 (81.2)	2 (66.7)	
Yes	49 (18.6)	23 (19.2)	26 (18.1)		5 (10.6)	44 (20.3)		48 (18.8)	1 (33.3)	

**Fig. 2:**
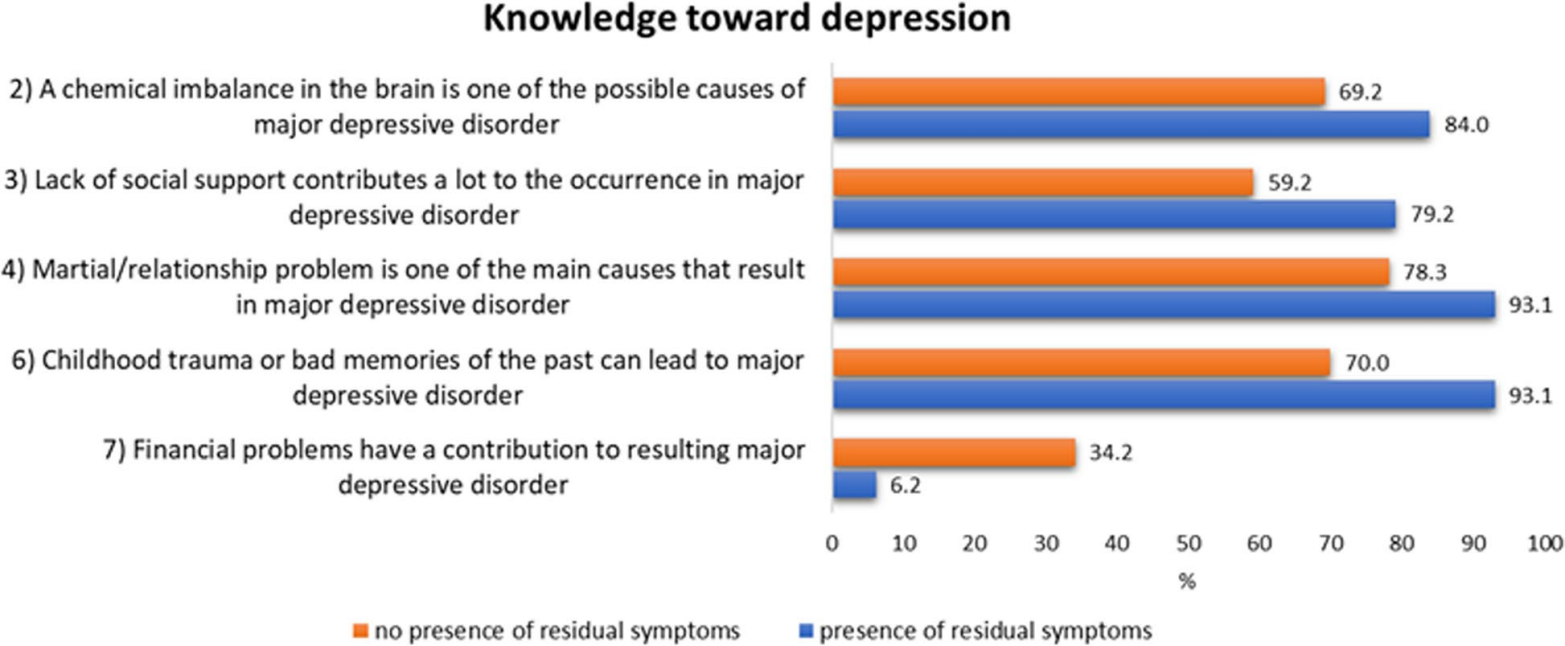
Statistical significance of knowledge between individuals with residual symptoms and those without residual symptoms of MDD (*p* < 0.01)

Additionally, it was found that there was a significant difference between the level of knowledge of MDD and the presence of residual symptoms of MDD. Individuals with MDD who reported residual symptoms had a higher level of knowledge of MDD (as measured by questions 2, 3, 4, 6, and 7) compared to those who did not report residual symptoms, as determined by a chi-square *p*-value of less than 0.001 (Fig. 2). However, no statistically significant difference was found between the attitudes toward MDD and the presence of residual symptoms of MDD (*p* > 0.05).

**Fig. 3:**
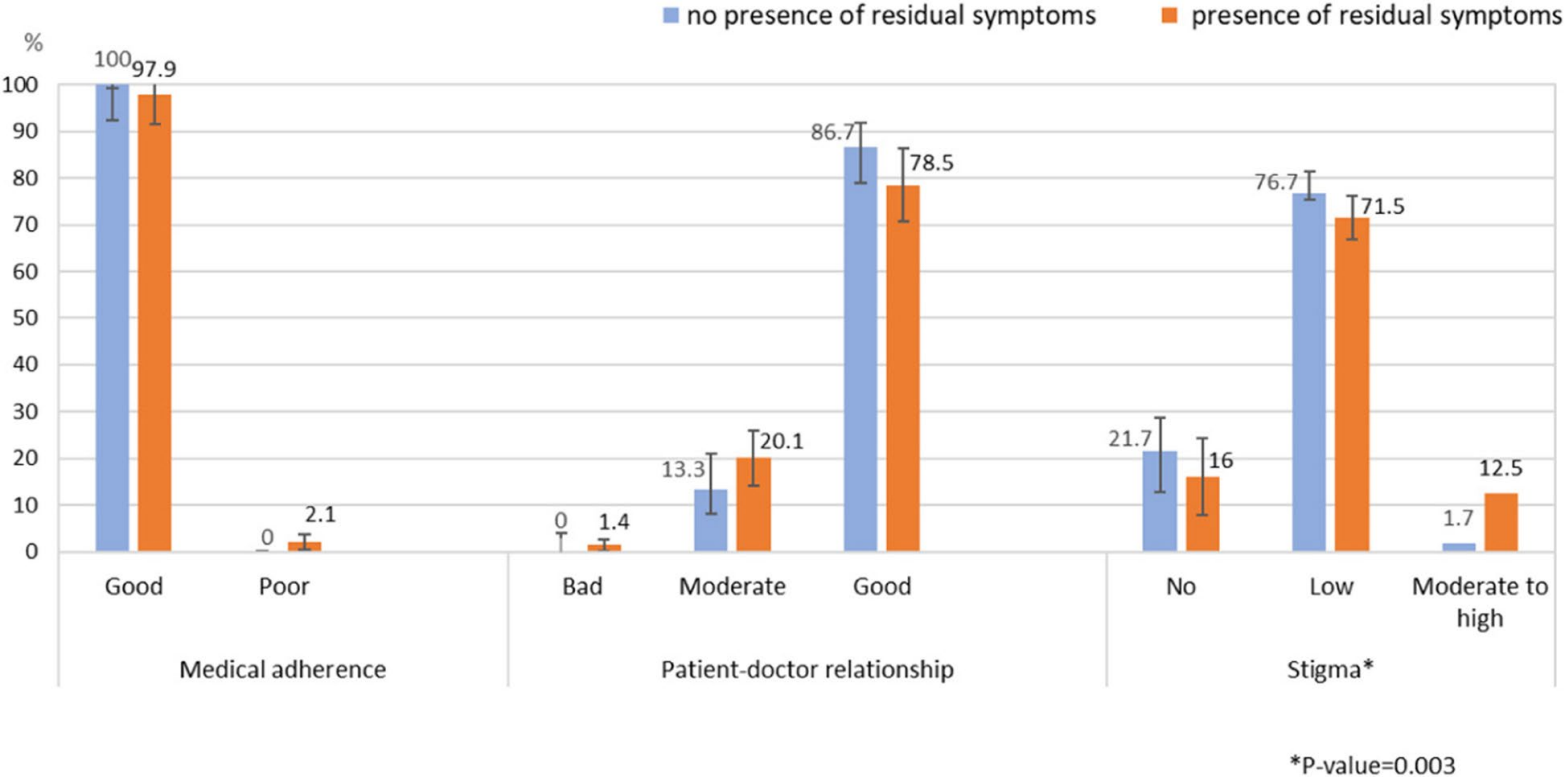
The association between the presence of residual symptoms of depression and level of stigma

Furthermore, when comparing the severity of MDD and the levels of stigma, it was found that individuals who reported moderately severe and severe depression had a higher proportion of stigma than other groups (Table 6). In addition, there was an association between the presence of residual symptoms of MDD and the level of stigma. The individuals with MDD who reported residual symptoms had a more moderate to a high level of stigma than individuals with MDD who reported no residual symptoms (12.5% vs. 1.7%, Chi-square *p*-value = 0.003) (Fig. 3). The most prevalent residual symptom of MDD was “feeling down”, which was statistically significantly associated with moderate level of stigma (*p*-value = 0.003).

**Table 6:** The association between level of depression by PHQ-9 score and level of stigma (*N* = 264)

Stigma level	Number (%)			
	PHQ-9 level			
	Minimal	Mild	Moderate	Moderately severe to severe
No	13 (19.1)	17 (26.6)	6 (11.5)	13 (16.2)
Low	53 (77.9)	47 (73.4)	41 (78.8)	54 (67.5)
Moderate	2 (2.9)	0 (0.0)	5 (9.6)	13 (16.2)

Note: Chi-square *p*-value = 0.003

**Fig. 4:**
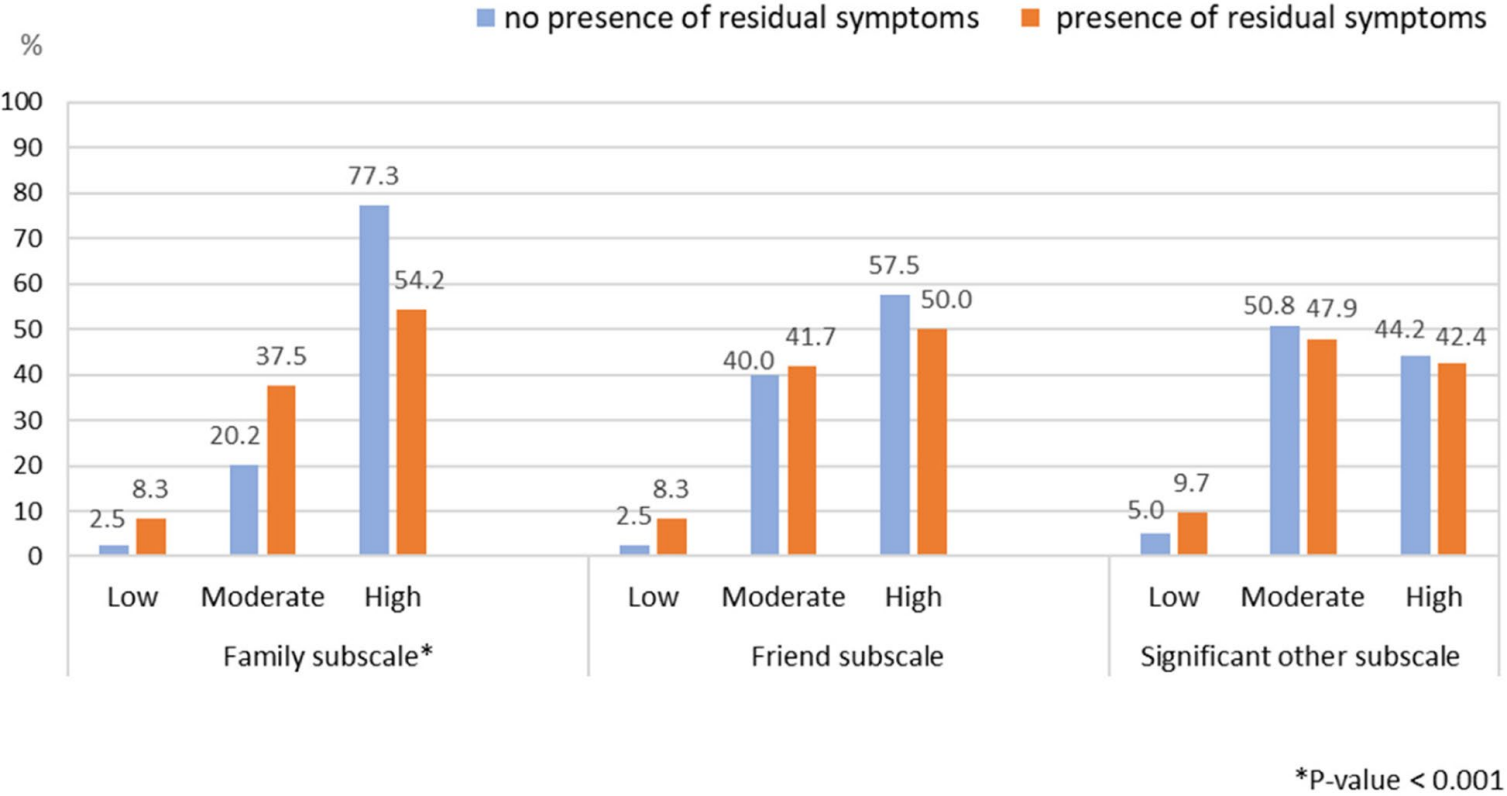
The association between the presence of residual symptoms of depression and perceived social support

In regards to the presence of residual symptoms of MDD and perceived social support, individuals with MDD who reported residual symptoms had a lesser level of perceived family support subscale vs. individuals with MDD who reported no residual symptoms (54.2% vs. 77.3%, Chi-square *p*-value < 0.001) (Fig. 4).

## Discussion

This is the first study from Southern Thailand that aimed to explore knowledge and attitudes toward MDD, as well as medication adherence, among individuals with MDD. Most participants reported a good level of awareness that marital or relationship problems, childhood trauma or bad memories, and chemical imbalance in the brain were some of the main causes of MDD. They disagreed with common stereotypical assumptions towards individuals with depression. The results of the study indicate that, while most participants had a high level of knowledge about MDD, there was a statistically significant difference between the level of knowledge and the presence of residual symptoms of MDD. Specifically, individuals with MDD who reported residual symptoms had a higher level of knowledge (as measured by questions 2, 3, 4, 6, and 7) compared to those who did not report residual symptoms. However, there was no statistically significant difference between the presence of residual symptoms of MDD and attitude towards MDD.

Most participants reported a low level or no stigma. Individuals who reported moderately severe and severe depression had a higher proportion of stigma compared to the other groups, and there was a correlation between the level of stigma and the presence of residual symptoms of MDD. The individuals with MDD who reported residual symptoms had a more moderate to a high level of stigma than individuals with MDD who reported no residual symptoms. Additionally, more than half of the participants reported a high level of perceived social support. However, individuals with MDD who reported residual symptoms had a lower level of perceived family support compared to those who did not report residual symptoms. This is likely because most participants reported having good medication adherence, therefore, this study could not indicate an association between the levels of knowledge and attitudes toward MDD with medication adherence.

Concerning medication adherence, these findings were different from those of prior reports that found that 60.0–70.0% of individuals with MDD had a high rate of medication non-adherence [7, 8, 19]. A potential explanation for these discrepancies may be due to different study instruments, and characteristics of the population; such as age differences. Our participants reported a mean age of 42.3 ± 18.3 years. A previous study found that older individuals with MDD, aged over 60 years old, reported lower medication adherence than younger individuals with MDD [19].

In regards to factors associated with medication non-adherence, a prior study indicated that one of the various factors was negative attitudes toward MDD [8], being distressed from being rejected or discriminated against, and feeling stigmatized [10]. However, this study indicated that most participants reported good medication adherence and no/low level of stigma including having a positive attitude toward MDD. They believed that individuals with MDD were not crazy or dangerous, these findings were different from a relevant prior study in UAE [25]. A potential explanation for these discrepancies may be the use of different study instruments and the characteristics of the population, as most of our participants were female and had a high level of education. Previous studies found that female gender and higher education are associated with better attitudes and beliefs towards antidepressants and MDD [13]. Additionally, it is also possible that these results reflect the efforts made by the Department of Psychiatry at the Faculty of Medicine, Prince of Songkla University, to increase public knowledge, reduce prejudiced attitudes or stigma, and promote positive attitudes towards all mental illnesses, including MDD.

A previous study from our Department of Psychiatry identified that most individuals with schizophrenia had good medication adherence and perceived their lives as meaningful [29]. Most of them, as well as their caregivers, perceived a low level of stigma [39] and a moderate quality of life [40]. However, stigmatization tended to be higher towards schizophrenia than depression [17, 18]. Nevertheless, among our study participants with MDD, most of them reported good medication adherence and a low level of stigma, including having a positive attitude toward MDD. Additionally, participants with residual symptoms of MDD were especially concerned with "feeling down", which was statistically significantly associated with a moderate level of stigma. This is potentially due to patients presenting with residual symptoms of "feeling down", feeling ashamed, or embarrassed (personal stigma). Therefore, perceived stigma together with personal stigma may have an impact on their interactions with people [41]. Consequently, if they believe or perceive that they are discriminated against by others, it could likely have a negative impact on their social support and environmental health [10, 41].

Additionally, providing a good level of knowledge and positive attitude toward MDD, having a good relationship between the physicians or health care team and the individuals with MDD, as per the finding in this study, may contribute to making individuals with MDD feel accepted by their physicians or others in their communities. It can subsequently create a positive outlook in connection to their self-esteem and illness or contribute to a more positive interpretation of MDD. These can result in better trust and treatment-related cooperation, resulting in lower medication non-adherence rates [42]. However, the results of the study indicate that there was a significant difference between the perceived relationship of family, having a physical illness, and the doctor-patient relationship. Therefore, the healthcare team should concern about these factors.

Concerning the presence of residual symptoms of MDD and the level of knowledge toward MDD, although most participants had a high level of knowledge toward MDD, the participants who reported residual symptoms of MDD had a higher level of knowledge than the participants who reported no residual symptoms of MDD. A potential explanation for these results may be that individuals with MDD who reported residual symptoms may have had the desire to search for more information and knowledge in order to explain their remaining symptoms. Therefore, they have a higher level of knowledge than individuals with MDD who have no residual symptoms. Furthermore, having a higher level of knowledge leads to a greater understanding of MDD, thus resulting in a positive attitude toward MDD. However, there was no significant difference in the attitudes toward MDD for both groups. These results provide new insight into understanding and addressing the needs of individuals with MDD who report residual symptoms.

In regards to the presence of residual symptoms of MDD and perceived family support, this study identified that participants who reported residual symptoms of MDD had less perceived family support than the participants who reported no residual symptoms of MDD. These findings are not different from a prior report that indicated that individuals with residual symptoms of MDD were associated with poor social support [16]. Nevertheless, the majority of participants reported a high level of social support. This may be due to cultural factors related to southern Thailand, as it is commonly believed that it has a strong sense of community and close-knit families that provide care and support to each other. Although our participants reported good medication adherence, the presence of residual symptoms of MDD was associated with some psychosocial factors. Therefore, in addition to encouraging regular medication use, providing psychoeducation, increasing knowledge, fostering a positive attitude, reducing stigma, implementing appropriate coping strategies, and ensuring social support for managing psychological distress should be the management of MDD. All of this will make the treatment of MDD more holistic [6].

Finally, some results were not part of the initial study objectives, but this study may provide valuable insights into the understanding and management of individuals with MDD including those who reported residual symptoms. Even though most participants were knowledgeable and had a good attitude toward MDD as well as cooperating in taking medicine, we found that more than half of the patients had residual symptoms of MDD. This was possible as a result of many causes such as unresolved symptoms, emotional bunting, insomnia disorder, or receiving antidepressants that did not cover all of the neurotransmitters that caused MDD [16, 22]. Therefore, physicians should be concerned about co-morbidity disorders, choose antidepressants by the symptoms of MDD, and be aware of the side effects of antidepressants. Moreover, the implementation of a national mental health policy to educate the public, promote positive attitudes, and reduce the stigma of MDD should be prioritized. The personal and perceived stigma towards MDD is an important issue that demands attention from public health as it can affect an individual's willingness to seek professional help. Public media campaigns should focus on increasing the credibility of the mental healthcare sector, but it is crucial to ensure that the content of these campaigns is adapted to the cultural norms of the targeted country [6].

To our knowledge, this was the only study, on this topic, in Southern Thailand during the past decade. However, this study was quantitative, and its sample size was prohibited to only MDD outpatients in lower Southern Thailand. Most participants were female in gender, had a moderate income, high educational level, and were in middle age groups. Hence, these results might not demonstrate the predicament or condition of all individuals with MDD for all genders, age groups, educational levels, economic statuses, and levels of disease severity including MDD inpatient or the whole country in a proportionate manner. Additionally, all participants were individuals with MDD who came to follow up regularly. But we did not collect data on the number of patient visits but only on treatment duration. Therefore, it might not cover individuals with MDD in all stages of illness, who did not follow up, and these patients may also feature poor medication adherence. However, this study attempted to collect data from all individuals with MDD and a review of scheduled appointments found that only a few of them missed their appointments, considered a low level of non-attendance. Even though there were no individuals with MDD who had poor medication adherence obtained from this study, we tried to search for the relationship between factors and the presence of residual symptoms of MDD. These findings may constitute novel knowledge that contains valuable information for the promotion of quality of life among individuals with MDD in the Southern Thai region. However, a more in-depth study is required on this subject. Henceforth, future studies should include a larger number of MDD outpatients with gender, age group, educational level, and economic status differences from other hospitals in Thailand; in other words, a multi-center study that purposes to identify this research topic should be employed. Moreover, such research should operate an in-depth methodology that is adept at analyzing specific factors or a more qualitative approach.

## Conclusion

Most participants reported good knowledge and a positive attitude toward depression. They exhibited good medication adherence, a low level of stigma, and a high level of social support. This study revealed a correlation between the presence of residual symptoms of depression and increased levels of knowledge, perceived stigma, and reduced family support.

## Data Availability

The qualitative data used in and analyzed during the current study cannot be made publicly available for confidentiality reasons, but they can be available on request from the corresponding author.

## Abbreviations

MAST: Medication adherence scale in Thai
MDD: Major depressive disorder
PDRQ-9: Patient-doctor relationship questionnaire
PHQ-9: Patient health questionnaire-9
rMSPSS: Revised Thai Multidimensional Scale of Perceived Social Support
